# Minimally invasive cardiac surgeries in 2022: annual report by Japanese Society of Minimally Invasive Cardiac Surgery

**DOI:** 10.1007/s11748-025-02225-z

**Published:** 2025-12-19

**Authors:** Tomoki Shimokawa, Hiraku Kumamaru, Noboru Motomura, Hiroyuki Nishi, Hiroyuki Nakajima, Hiroyuki Kamiya, Kazuma Okamoto, Soh Hosoba, Yoshikatsu Saiki, Takashi Miura, Minoru Tabata, Akira Shiose, Taichi Sakaguchi

**Affiliations:** 1 Scientific Registry Committee of Japanese Society of Minimally Invasive Cardiac Surgery, Tokyo, Japan; 2 Japan Cardiovascular Surgery Database Organization, Tokyo, Japan; 3 Department of Cardiovascular Surgery, Teikyo University, Tokyo, Japan; 4 Department of Healthcare Quality Assessment, The University of Tokyo, Tokyo, Japan; 5 Department of Cardiovascular Surgery, Toho University Sakura Medical Center, Chiba, Japan; 6 Department of Cardiovascular Surgery, Tokai University Hachioji Hospital, Tokyo, Japan; 7 Department of Cardiovascular Surgery, Akita University, Akita, Japan; 8 Department of Cardiovascular Surgery, Asahikawa Medical University, Hokkaido, Japan; 9 Department of Cardiovascular Surgery, Hamamatsu University School of Medicine, Shizuoka, Japan; 10 Department of Cardiovascular Surgery, Nagoya Tokushukai General Hospital, Aichi, Japan; 11 Department of Cardiovascular Surgery, Tohoku University Graduate School of Medicine, Miyagi, Japan; 12 Department of Cardiovascular Surgery, Nagasaki University, Nagasaki, Japan; 13 Department of Cardiovascular Surgery, Juntendo University, Tokyo, Japan; 14 Department of Cardiovascular Surgery, Kyushu University, Fukuoka, Japan; 15 Department of Cardiovascular Surgery, Hyogo Medical College, Hyogo, Japan

**Keywords:** Annual report, Minimally invasive cardiac surgery, Japanese society of minimally invasive cardiac surgery

## Abstract

**Purpose:**

Up-to-date national data on minimally invasive cardiac surgery (MICS) are essential for quality control but remain limited. This report summarizes 2022 outcomes of right- or left-minithoracotomy, thoracoscopic/port-assisted, and robotic-assisted MICS in Japan, based on the Japan Cardiovascular Surgery Database (JCVSD).

**Methods:**

Data were collected from patients undergoing mitral valve repair/replacement (MV repair, *n* = 2525; MVR, *n* = 279), aortic valve replacement (AVR, *n* = 1114), coronary artery bypass grafting (CABG, *n* = 450), atrial septal defect closure (ASD, *n* = 212), and cardiac tumor resection (*n* = 113) using MICS approaches. Perioperative data included 30-day and in-hospital mortality, conversion rates, and major morbidities.

**Results:**

For MV repair, the 30-day and in-hospital mortality rates were 0.3% and 0.2% in isolated cases (*n* = 1461) and 0.4% and 0.6% overall, respectively. Mortality rates for MVR were 2.5% and 4.2% in isolated cases (*n* = 120) and 2.9% and 4.3% overall, respectively. Mortality rates for AVR were 0.6% and 1.0% in isolated cases (*n* = 981) and 0.9% and 1.3% overall, respectively. Mortality rates for CABG were 1.6% and 1.8%, respectively. Mortality rates were 0% for both ASD closure and tumor resection. Across the groups, conversion to full sternotomy ranged from 0% to 1.8%.

**Conclusion:**

The 2022 nationwide MICS data demonstrate consistently low mortality and morbidity across all procedure types. As MICS adoption grows, these updated JCVSD findings will serve as vital benchmarks for ongoing quality improvement in Japan.

## Introduction

As a minimally invasive cardiac surgery (MICS) procedure, open heart surgery using minithoracotomy has recently become widespread worldwide [1, 2], with several studies reporting its safety [3, 4]. On the other hand, it is true that there are some opposing opinions on MICS surgery [5], and issues such as the learning curve [6] and prolonged operation time [1–8] have long been pointed out. In Japan, public health insurance reimbursement for valve surgery using a sternal preservation method began in 2018, resulting in an increasing number of institutions performing MICS valve surgery with minithoracotomy [9]. Thus, reevaluation of the procedure is necessary.

Since its establishment in 2015, the Japanese Society for Minimally Invasive Cardiac Surgery (J-MICS) has been working towards the safe spread of MICS surgery as one of its missions. In light of the increasing use of MICS procedures, it was decided to examine and present findings from the annual report of MICS surgical procedures starting in 2021, in recognition of the importance of clarifying real-world data at a national level. This study presents findings from an investigation of the 2021 annual report [9] related to the mitral valve (MV), aortic valve, coronary artery bypass grafting (CABG), atrial septal defect (ASD) closure, and cardiac tumor resection procedures using data from the Japan Cardiovascular Surgery Database (JCVSD), a nationwide registry of clinical information from nearly all hospitals that perform cardiovascular surgery in Japan.

## Patients and methods

### Study population

The records of patients who underwent MV surgery, AVR, CABG, ASD closure, or resection of a cardiac tumor via right or left minithoracotomy, or thoracoscopic/port-assisted, or robotic-assisted approaches between January 1 and December 31, 2022, were obtained from the JCVSD [10]. Data were collected using the National Clinical Database platform. The dataset includes approximately 300 variables (definitions available online at http://www.jacvsd.umin.jp), consistent with those included in the Society of Thoracic Surgeons (STS) National Database (definitions available online at http://sts.org), which includes information on the surgical approach. Data registration and use for research purposes were approved by the institutional review board of the National Clinical Database (16–041, Aug. 29, 2016). The accuracy of the submitted data was verified by random monthly visits to each hospital by the site visit working group [11].

A total of 61,606 cardiovascular surgeries performed in 2022 were registered in the JCVSD. Among the 17,260 patients with valvular heart disease, 3918 underwent a right-lateral thoracotomy or a robotic approach. This MICS subset comprised 2804 MV cases and 1114 aortic valve cases. Patients who underwent partial sternotomy (upper or lower hemi-sternotomy) or left thoracotomy were excluded, and patients who underwent procedures without aortic cross-clamping, including those requiring circulatory arrest or ventricular fibrillation, were also excluded. The MV cohort included combined procedures, including tricuspid valve surgery, rhythm procedures such as the Maze procedure or pulmonary vein isolation, and aortic valve surgery. To provide context, national totals (all approaches) were 8848 MV procedures and 9747 AVR procedures in 2022. Accordingly, estimated MICS penetration was 31.7% for MV surgery (2804/8848) and 11.4% for AVR (1114/9747). These totals are cited from the JATS annual report [12] and/or JCVSD aggregates. Among the registered cases, there were 14,656 CABG cases, 777 ASD cases, and 634 cardiac tumor cases in 2022. From these cohorts, cases using a left lateral approach for CABG and a right lateral approach for ASD and tumor procedures were included. Cases that did not match the MICS approach involving left or right minithoracotomy were excluded. The study population flowchart is presented in the 2021 annual report [9].

#### Study design

Preoperative variables and early postoperative outcome definitions, including those previously defined by the STS, were used. Thirty-day mortality was defined as death within 30 days of surgery, regardless of the geographic location of the patient, including discharge from the hospital. In-hospital mortality was defined as death within any time interval following surgery in a patient who was not discharged from the hospital. Hospitalization was defined as the postoperative length of stay. A median sternotomy conversion case was one in which port access was not completed, with a right or left lateral approach and median approach selected in the JCVSD. The distribution of the number of MV surgery cases per institution was also evaluated. JapanSCORE was used to evaluate the distribution of predicted mortality among patients.

## Results

### Mitral valve surgery

The MICS-MV procedures included 2525 repairs and 279 replacements; of these, 1461 (57.9%) were isolated repairs and 120 (43.0%) were isolated replacements (Table 1). The mean age ± standard deviation of the patients was 59.0 ± 12.8 years for isolated MV repair, 61.7 ± 12.9 years for total MV repair cases, 65.3 ± 13.4 years for isolated MVR, and 69.3 ± 12.2 years for total MVR cases. Of these, 73 (2.8%) MV repairs and 45 (16.1%) replacements were performed on an urgent or emergency basis. Infective endocarditis was noted in 119 (4.7%) MV repair cases and 38 (13.6%) MVR cases. The JapanSCORE was 1.0 ± 1.4% for isolated MV repair and 6.7 ± 11.4% for isolated MVR. Concomitant procedures included tricuspid valve surgery, consisting of MV repair in 503 (19.9%) cases and replacement in 96 (34.4%) cases; arrhythmia surgery, consisting of MV repair in 552 (21.9%) cases, replacement in 85 (30.5%) cases; AVR, MV repair in 32 (1.3%) cases, and replacement in 11 (3.9%) cases.

**Table 1:** Preoperative and intraoperative characteristics of patients who underwent mitral valve surgery

	Mitral valve repair		Mitral valve replacement	
	Total (n = 2525)	Isolated (n = 1461)	Total (n = 279)	Isolated (n = 120)
Age (years)	61.7 ± 12.9	59.0 ± 12.8	69.3 ± 12.2	65.3 ± 13.4
Sex (Male/Female)	1552/973	924/537	125/154	64/56
Body surface area (m^2^)	1.65 ± 0.20	1.66 ± 0.20	1.55 ± 0.20	1.59 ± 0.23
Dialysis	18 (0.7%)	3 (0.2%)	10 (3.6%)	5 (4.2%)
Infective endocarditis (Active/treated)	119 (4.7%) (41/78)	86 (5.9%) (31/55)	38 (13.6%) (29/9)	29 (24.2%) (27/2)
Urgent/emergent	68/5	36/5	43/2	33/2
Japan Score (%)	–	1.0 ± 1.4	–	6.7 ± 11.4
Operation time (min)	281 ± 103	262 ± 96	319 ± 115	318 ± 133
CPB time (min)	183 ± 78	169 ± 75	208 ± 88	206 ± 103
Cardiac arrest time (min)	122 ± 52	111 ± 47	137 ± 57	130 ± 63
Transfusion	1103 (43.7%)	558 (38.2%)	202 (72.4%)	88 (73.3%)
IABP/PCPS	11/12	2/5	5/5	3/3
Tricuspid valve surgery	503 (19.9%)	–	96 (34.4%)	–
Arrhythmia surgery (MAZE/PVI/ LAAC)	552 (21.9%) (289/63/472)	–	85 (30.5%) (39/8/70)	–
Aortic valve surgery	32 (1.3%)	–	11 (3.9%)	–
Conversion	16 (0.6%)	8 (0.5%)	2 (0.7%)	2 (1.7%)

CPB, cardiopulmonary bypass; IABP, intraaortic balloon pumping; PCPS, percutaneous cardiopulmonary system; PVI, pulmonary vein isolation; LAAC, left atrium appendage closure

The operative time was 262 ± 96 min for isolated MV repair, 281 ± 103 min for total MV repair, 318 ± 133 min for isolated MVR, and 319 ± 115 min for total MVR cases. Conversion to median sternotomy was noted in 16 (0.6%) cases of MV repair and in 2 (0.7%) cases of MVR.

The 30-day and in-hospital mortality rates were 0.3 and 0.2%, respectively, for isolated MV repair and 0.4 and 0.6%, respectively, for total MV repair cases (Table 2). In MVR, the 30-day and in-hospital mortality rates were 2.5 and 4.2%, respectively for isolated replacement cases and 2.9% and 4.3% for total MVR cases. Re-exploration for bleeding was performed in 1.3% of total MV repair cases and 4.7% of total MVR cases. Stroke occurred in 0.8% of total MV repair cases and 4.3% of total MVR cases. The rates of myocardial infarction were below 1.0% for MV repair cases and approximately 1.0% for MVR cases. No cases of aortic dissection were observed. Leg ischemia occurred in less than 1.0% of cases in both groups. For total MV repair patients, the median ICU stay was 2 days and the median hospital stay was 11 days, while median ICU and hospital stay for total MVR patients were 3 and 15 days, respectively.

**Table 2:** Postoperative results of patients who underwent mitral valve surgery

		Mitral valve repair			Mitral valve replacement	
	Total (n = 2525)		Isolated (n = 1461)	Total (n = 279)		Isolated (n = 120)
30-day mortality	11 (0.4%)		4 (0.3%)	8 (2.9%)		3 (2.5%)
In hospital mortality	15 (0.6%)		3 (0.2%)	12 (4.3%)		5 (4.2%)
Reoperation for bleeding	33 (1.3%)		19 (1.3%)	13 (4.7%)		4 (3.3%)
Stroke	21 (0.8%)		9 (0.6%)	12 (4.3%)		2 (1.7%)
Thoracotomy infection	4 (0.2%)		3 (0.2%)	0		0
Groin area infection	9 (0.4%)		4 (0.3%)	1 (0.4%)		0
Prolonged ventilation	38 (1.5%)		8 (0.5%)	20 (7.2%)		8 (6.7%)
Renal failure	45 (1.8%)		12 (0.8%)	20 (7.2%)		13 (10.8%)
New onset of AF	315 (12.5%)		144 (9.9%)	46 (16.5%)		18 (15%)
Heart block	24 (1%)		12 (0.8%)	4 (1.4%)		3 (2.5%)
Perioperative MI	7 (0.28%)		4 (0.3%)	3 (1.1%)		2 (1.7%)
Aortic dissection	0		0	0		0
Acute limb ischemia	2 (0.1%)		1 (0.1%)	0		0
ICU stay (days)	2 (median)		2 (median)	3 (median)		3 (median)
Hospital stay (days)	11(median)		10(median)	15(median)		15(median)

AF, atrial fibrillation; MI, myocardium infarction; ICU, intensive care unit

**Fig.1:**
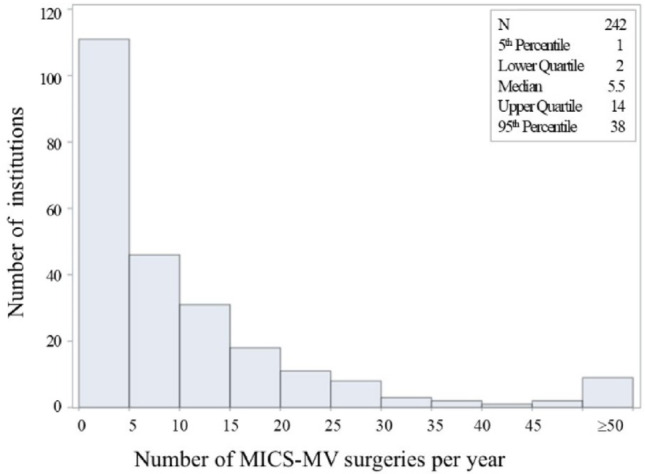
Distribution of the numbers of MICS-mitral cases among medical institutions. Several institutions treated fewer than 10 cases per year

The distribution of the number of MICS-MV procedures performed at each hospital was shown in Fig. 1. Approximately 111 facilities had fewer than 5 cases per year, while nearly 85% had fewer than 10 cases annually. However, several facilities were found to treat more than 50 cases per year.

### Aortic valve replacement

The mean age was 68.6 ± 12.3 years for patients with isolated AVR and 68.9 ± 12.1 years for total AVR patients (Table 3). Of these, 28 (2.5%) patients underwent AVR on an urgent or emergency basis. There were 23 (2.3%) cases of infective endocarditis among isolated AVR patients and 27 (2.4%) cases among total AVR patients. The JapanSCORE was 2.7 ± 2.9% in isolated AVR. Concomitant procedures included tricuspid valve surgery (n = 15, 1.3%) and arrhythmia surgery (n = 46, 4.1%), with operation times of 252 ± 92 min for isolated AVR and 260 ± 100 min for total AVR cases. Conversion to median sternotomy was performed in 12 (1.2%) isolated AVR cases and 18 patients (1.6%) among total AVR cases.

**Table 3:** Preoperative and intraoperative characteristics of patients who underwent aortic valve surgery

	Total (n = 1114)	Isolated (n = 981)
Age (years)	68.9 ± 12.1	68.6 ± 12.3
Sex (Male/Female)	681/433	599/382
Body surface area (m^2^)	1.63 ± 0.2	1.63 ± 0.2
Dialysis	53 (4.8%)	44 (4.5%)
Infective endocarditis (Active/treated)	27 (2.4%) (10/17)	23 (2.3%) (8/15)
Urgent/emergent	26/2	17/2
Japan Score (average, %)	–	2.7 ± 2.9
Operation time (min)	260 ± 100	252 ± 92
CPB time (min)	154 ± 68	149 ± 60
Cardiac arrest time (min)	104 ± 46	100 ± 41
Mechanical/tissue valve/others	118/983/13	107/864/10
Transfusion	633 (56.8%)	546 (55.7%)
IABP/PCPS	10/8	3/4
Tricuspid valve surgery	15 (1.3%)	–
Arrhythmia surgery (MAZE/PVI/LAAC)	46 (4.1%) (8/5/38)	–
Conversion	18 (1.6%)	12 (1.2%)

CPB, cardiopulmonary bypass; IABP, intraaortic balloon pumping; PCPS, percutaneous cardiopulmonary system; PVI, pulmonary vein isolation; LAAC, left atrium appendage closure

The 30-day and in-hospital mortality rates for isolated AVR cases were 0.6 and 1.0%, respectively, while those for total AVR cases were 0.9 and 1.3%, respectively (Table 4). Re-exploration for bleeding was performed in 22 (2.0%) patients. The rate of stroke occurrence was approximately 1%, with no cases affected by myocardial infarction, while aortic dissection and lower limb ischemia were observed in one (0.1%) patient each. The median ICU stay was 2 days and the overall hospital stay was 12 days.

**Table 4:** Postoperative results of patients who underwent aortic valve replacement

	Total (n = 1114)	Isolated (n = 981)
30-day mortality	10 (0.9%)	6 (0.6%)
In hospital mortality	14 (1.3%)	10 (1.0%)
Reoperation for bleeding	22 (2.0%)	20 (2.0%)
Stroke	14 (1.3%)	12 (1.2%)
Thoracotomy infection	0	0
Groin area infection	2 (0.2%)	1 (0.1%)
Prolonged ventilation	16 (1.4%)	11 (1.1%)
Renal failure	21 (1.9%)	17 (1.7%)
New onset of AF	175 (15.7%)	143 (14.6%)
Heart block	22 (2.0%)	18 (1.8%)
Perioperative MI	0	0
Aortic dissection	1 (0.1%)	1 (0.1%)
Acute limb ischemia	1 (0.1%)	1 (0.1%)
ICU stay (days)	2 (median)	2 (median)
Hospital stay (days)	12 (median)	11 (median)

AF, atrial fibrillation; MI, myocardial infarction; ICU, intensive care unit

### Coronary artery bypass grafting

In total, 450 MICS-CABG procedures were performed (Table 5). There were 137 patients with left main trunk lesions, 150 with 3-vessel disease, and 131 with single-vessel disease. Off-pump CABG was performed in 423 (94.0%) patients and single-vessel bypass was performed in 315 (70.0%) patients. The left internal thoracic artery was used in 421 cases (93.6%), whereas the right internal thoracic artery, radial artery, saphenous vein, and gastroepiploic artery were used in 45, 28, 82, and 23 cases, respectively. There were 7 (1.6%) conversion cases.

**Table 5:** Preoperative and intraoperative characteristics of patients who underwent MICS-CABG

	Isolated (n = 450)
Age (years)	71.0 ± 10.7
Gender (Male/Female)	351/99
Body surface area (m^2^)	1.65 ± 0.2
Dialysis	47 (10.7%)
Urgent/emergent	62/3
Japan score (average, %)	2.8 ± 4.3
Operation time (min)	234 ± 108
Transfusion	123 (27.3%)
IABP/PCPS	24/4
Japan score (average, %)	2.8 ± 4.3
Number of diseased vessels	1VD:131, 2VD:157, 3VD:150, LMT:137, Unknown:12
Off pump versus on pump	Off pump: 423, On pump: 27
Number of distal anastomoses	1: 315, 2: 77, 3: 47, 4: 9, 5: 1, 6: 1
Bypass graft	LITA: 421, SV: 82, RITA: 45, RA: 28, GEA: 23
Conversion	7 (1.6%)

VD, vessel disease; ITA, internal thoracic artery; SV, saphenous vein; GEA, gastroepiploic artery; RA, radial artery

The 30-day and in-hospital mortality rates were 1.6 and 1.8%, respectively, and 1.1% of the patients underwent re-exploration for bleeding (Table 5). The rate of stroke was < 1%, and no cases of myocardial infarction were noted. The median ICU stay was 2 days and the median hospital stay was 10 days (Table 6).

**Table 6:** Postoperative results of patients who underwent MICS-CABG

	Isolated (n = 450)
30-day mortality	7 (1.6%)
In hospital mortality	8 (1.8%)
Reoperation for bleeding	5 (1.1%)
Stroke	2 (0.4%)
Prolonged ventilation	5 (1.1%)
Renal failure	7 (1.6%)
New onset of AF	35 (7.8%)
PMI	0
ICU stay (days)	2 (median)
Hospital stay (days)	10 (median)

AF, atrial fibrillation; MI, myocardium infarction; ICU, intensive care unit

### Atrial septal defect and cardiac tumors

The mean age of the ASD patients was 54.2 ± 19.8 years, while that of patients treated for cardiac tumors was 65.6 ± 15.2 years (Table 7). Eighty-four (74.3%) of the cardiac tumor cases involved myxoma. Concomitant procedures included tricuspid valve surgery (ASD, n = 79, 37.3%; cardiac tumor, n = 3, 2.7%) and arrhythmia surgery (ASD, n = 45, 22.3%; cardiac tumor, n = 8, 7.1%). The operation times for the ASD and cardiac tumor cases were 263 ± 87 and 230 ± 85 min, respectively. Conversion to a median sternotomy was noted in none of the ASD cases and in 2 (1.8%) of the cardiac tumor cases.

**Table 7:** Preoperative and intraoperative characteristics of patients with atrial septum defect and cardiac tumors

	ASD (n = 212)	Cardiac tumor (n = 113)
Age (years)	54.2 ± 19.8	65.6 ± 15.2
Sex (Male/Female)	90/122	36/77
Body surface area (m^2^)	1.61 ± 0.19	1.56 ± 0.20
Dialysis	0	2 (1, 8%)
Infective endocarditis (Active/treated)	5 (1.4%) (3/2)	0
Urgent/emergent	3/0	16/2
Japan Score	–	–
Operation time (min)	263 ± 87	230 ± 85
CPB time (min)	160 ± 63	128 ± 62
Arrest time(min)	99 ± 48	72 ± 38
Transfusion	77 (36.3%)	61 (54%)
IABP/PCPS	0/1	0/0
Tricuspid valve surgery	79 (37.3%)	3 (2.7%)
Arrhythmia surgery (MAZE/PVI/LAAC)	45 (21.2%) (25/7/35)	8 (7.1%) (6/0/6)
Myxoma	–	84 (74.3%)
Conversion	0	2 (1.8%)

CPB, cardiopulmonary bypass; IABP, intraaortic balloon pumping; PCPS, percutaneous cardiopulmonary system; PVI, pulmonary vein isolation; LAAC, left atrium appendage closure

For ASD cases, both the 30-day and in-hospital mortality rates were 0%, and for cardiac tumor cases, both rates were also 0% (Table 8). Re-exploration for bleeding was performed in 1.4% of the ASD cases and in none of the cardiac tumor cases. The rate of stroke was 1.4% in the ASD cases and 3.5% in the cardiac tumor cases. The median ICU stay for ASD cases was 2 days and the median hospital stay was 10 days, whereas the median ICU and hospital stay for cardiac tumor cases were 2 and 11 days, respectively.

**Table 8:** Postoperative results of patients with atrial septum defect and cardiac tumors

	ASD (n = 212)	Cardiac tumor (n = 113)
30-day mortality	0	0
In hospital mortality	0	0
Reoperation for bleeding	3 (1.4%)	0
Stroke	3 (1.4%)	4 (3.5%)
Thoracotomy infection	1 (0.5%)	0
Groin area infection	0	0
Prolonged ventilation	3 (1.4%)	1 (0.9%)
Renal failure	1 (0.5%)	0
New onset of AF	30 (14.2%)	17 (15%)
PMI	0	0
Aortic dissection	0	0
Acute limb ischemia	0	0
ICU stay (days)	2 (median)	2 (median)
Hospital stay (days)	10 (median)	11 (median)

AF, atrial fibrillation; MI, myocardium infarction; ICU, intensive care unit

## Discussion

This nationwide report compiles the perioperative outcomes of minithoracotomy-based MICS in Japan for 2022. Building on the 2021 findings, it delivers updated, comprehensive data across all major MICS categories (MV repair and replacement, AVR, coronary artery bypass grafting, atrial septal defect closure, and cardiac tumor resection) sourced from institutions nationwide. The timely dissemination of these results supports ongoing quality assurance and benchmarking in clinical practice. As MICS adoption continues to grow, these 2022 outcomes offer vital insights into procedural safety, efficacy, and institutional performance, guiding best practices and driving further advances in minimally invasive cardiac surgery.

For isolated MV repair, the most common MICS procedure, the 30-day mortality and in-hospital mortality rates were 0.3 and 0.2%, respectively. For reference, the rates in 2021 were 0.1 and 0.2%. Although no results are available for direct comparison, the annual report of the Japanese Association for Thoracic Surgery (JATS) presented in 2022 [12] showed that the 30-day mortality rate for isolated MV repair was 0.7%, while the in-hospital mortality rate was 1.1%. According to the 2022 Annual Report of the JATS, isolated MVR results in 30-day and in-hospital mortality rates of 4.1 and 7.3%, respectively. In contrast, MICS-MVR in Japan achieved lower rates of 2.5 and 4.2% in 2022, compared with 4.7 and 4.7% in 2021, indicating consistently low mortality and a favorable safety profile. In addition, although many hospitals performed only a small number of MICS procedures annually, mirroring the low‐volume trend seen in the STS database report [13], the number of institutions reporting 1–4 mitral MICS cases per year was greater in 2022 than in 2021. Despite this growth in low-volume centers, overall surgical outcomes remained stable. Although the results may still be influenced by several high-volume institutions, these data further support the validity and robustness of the MICS approach in Japan. Unfortunately, specific data on the number of robot-assisted MICS procedures in 2022 are not available; however, the perioperative outcomes of robot-assisted approaches have been reported to be comparable to those of conventional minithoracotomy-based MICS in Japan [14].

This report also summarizes minimally invasive aortic valve surgeries performed without median sternotomy, excluding any cases that involved partial sternotomy. Isolated AVR via MICS in 2022 had 30-day mortality and in-hospital mortality rates of 0.6% and 1.0%, respectively, which were substantially lower than the rates of 1.5% and 2.5% in the 2022 annual report of the JATS. These values also align closely with the 2021 outcomes (30-day mortality, 0.5%; in-hospital mortality, 0.5%).

The number of MICS-CABG procedures increased from 400 cases in 2021 to 450 in 2022, with off-pump CABG performed in 423 patients (94.0%) and single-vessel bypass performed in 315 patients (70.0%). The left internal thoracic artery was used in 421 (93.6%) patients. A multivessel bypass procedure was performed in 135 (30.0%) cases, up from 23.0% in 2021, indicating the selection of this approach for more complex diseases. The 30-day and in-hospital mortality rates were 1.6 and 1.8%, respectively, slightly above the 2021 rates of 0.8 and 0.5%, and exceeded the off-pump CABG benchmarks of 1.1 and 1.5% described in the 2022 annual report of the JATS. Conversion to full sternotomy increased in 2022, and more patients underwent multivessel bypass, both of which could increase mortality. Future analyses should include direct comparisons with established risk scores to clarify the risk profile for MICS-CABG.

Finally, among ASD and cardiac tumor cases via right minithoracotomy, 2022 outcomes showed concurrent tricuspid repair in 37.3% of ASD patients and myxoma in 74.3% of tumor cases. The 30-day and in-hospital mortality rates were both 0%, equivalent to the overall outcomes in the annual report of the JATS during 2022, which showed 30-day and in-hospital mortality rates of 0 and 0.1%, respectively, for ASD and 1.1 and 1.8% for cardiac tumor cases.

### Limitations

This study is associated with several limitations. First, the JCVSD lacked certain MICS-specific variables for 2022, preventing the analysis of important factors such as the technical quality of MV repair or the incidence of intraoperative conversion from repair to replacement. Likewise, complications unique to MICS could not be identified. Serious vascular complications associated with femoral arterial or venous cannulation were not observed.

Second, although we benchmarked MICS outcomes against the JATS annual report, comprehensive 2022 data for conventional (non-MICS) cohorts, particularly detailed JapanSCORE distributions, are not uniformly available. Consequently, precise comparisons of JapanSCORE risk profiles between MICS and non-MICS cannot be performed for all procedure types at this time. Where reliable non-MICS figures exist, we have included them for context and explicitly indicated when cross-cohort comparisons are not feasible. We plan to incorporate full non-MICS risk profiles once detailed national data become available.

Third, the registry did not include granular information on the use of robot assistance in MICS, the underlying etiology or pathophysiology of MV disease, the histopathological subtypes of cardiac tumors, or the extent and handling of missing data (e.g., imputation methods).

To address some of these gaps, J-MICS and the JCVSD collaborated to introduce 20 additional perioperative parameters specific to MICS, beginning in 2024. These enhancements will enable more detailed safety and efficacy analyses in future nationwide reports.

## Abbreviations

ASD: Atrial Septal Defect
AVR: Aortic Valve Replacement
CABG: Coronary Artery Bypass Grafting
JATS: Japanese Association for Thoracic Surgery
JCVSD: Japanese Cardiovascular Surgery Database
J-MICS: Japanese Society for Minimally Invasive Cardiac Surgery
MICS: Minimally Invasive Cardiac Surgery
MV: Mitral Valve
MVR: Mitral Valve Replacement
STS-PROM: Society of Thoracic Surgeons Predicted Risk of Mortality
